# Positive feedback between lncRNA FLVCR1-AS1 and KLF10 may inhibit pancreatic cancer progression via the PTEN/AKT pathway

**DOI:** 10.1186/s13046-021-02097-0

**Published:** 2021-10-11

**Authors:** Jiewei Lin, Shuyu Zhai, Siyi Zou, Zhiwei Xu, Jun Zhang, Lingxi Jiang, Xiaxing Deng, Hao Chen, Chenghong Peng, Jiaqiang Zhang, Baiyong Shen

**Affiliations:** 1 Department of General Surgery, Pancreatic Disease Center, Research Institute of Pancreatic Diseases, Ruijin Hospital, Shanghai Jiao Tong University School of Medicine, Shanghai, China; 2 Research Institute of Pancreatic Diseases, Shanghai Jiao Tong University School of Medicine, Shanghai, China; 3 State Key Laboratory of Oncogenes and Related Genes, Shanghai, China; 4 Institute of Translational Medicine, Shanghai Jiao Tong University, Shanghai, China

**Keywords:** lncRNA, FLVCR1-AS1, KLF10, Pancreatic cancer, ceRNA

## Abstract

**Background:**

FLVCR1-AS1 is a key regulator of cancer progression. However, the biological functions and underlying molecular mechanisms of pancreatic cancer (PC) remain unknown.

**Methods:**

FLVCR1-AS1 expression levels in 77 PC tissues and matched non-tumor tissues were analyzed by qRT-PCR. Moreover, the role of FLVCR1-AS1 in PC cell proliferation, cell cycle, and migration was verified via functional *in vitro* and *in vivo* experiments. Further, the potential competitive endogenous RNA (ceRNA) network between FLVCR1-AS1 and KLF10, as well as FLVCR1-AS1 transcription levels, were investigated.

**Results:**

FLVCR1-AS1 expression was low in both PC tissues and PC cell lines, and FLVCR1-AS1 downregulation was associated with a worse prognosis in patients with PC. Functional experiments demonstrated that FLVCR1-AS1 overexpression significantly suppressed PC cell proliferation, cell cycle, and migration both *in vitro* and *in vivo*. Mechanistic investigations revealed that FLVCR1-AS1 acts as a ceRNA to sequester miR-513c-5p or miR-514b-5p from the sponging KLF10 mRNA, thereby relieving their suppressive effects on KLF10 expression. Additionally, FLVCR1-AS1 was shown to be a direct transcriptional target of KLF10.

**Conclusions:**

Our research suggests that FLVCR1-AS1 plays a tumor-suppressive role in PC by inhibiting proliferation, cell cycle, and migration through a positive feedback loop with KLF10, thereby providing a novel therapeutic strategy for PC treatment.

**Supplementary Information:**

The online version contains supplementary material available at 10.1186/s13046-021-02097-0.

## Background

Pancreatic cancer (PC) is one of the most lethal digestive tract malignancies, characterized by late diagnosis, grave prognosis, high metastasis rates, and a 5-year survival rate of < 10 % [1, 2]. Moreover, due to low radical surgery rates and high recurrence risks, treatment options are severely limited. Thus, chemotherapy, primarily gemcitabine and gemcitabine-based comprehensive treatments, remains indispensable for advanced PC patients despite widespread chemotherapeutic resistance [3]. Thus far, few functional therapeutic targets for PC have been established. Hence, investigation of the underlying molecular mechanisms of PC tumorigenesis is imperative to uncover novel therapeutic targets.

Recent advances in RNA sequencing technology have revealed that the majority of human DNA is actively transcribed, but only approximately 2 % of transcripts are able to encode proteins, while most human genome transcripts are non-coding RNAs [4]. Recent studies have focused on the role of long non-coding RNAs (lncRNAs) in cancer, as lncRNAs play an important role in cell proliferation, cell cycle, cell migration, apoptosis, and aerobic glycolysis [5, 6]. Additionally, various mechanisms of action have been identified, including transcriptional modulation, protein translation, protein function, and RNA stability [7, 8]. Multiple lncRNAs have been shown to be key regulators of PC tumorigenesis [9, 10], but their molecular functions in PC remain unknown. Thus, there is an urgent need to elucidate the molecular mechanisms of lncRNAs in PC pathogenesis. The biological functions of lncRNAs are determined by their subcellular localization [11]. Intranuclear lncRNAs can be directly involved in transcriptional regulation, whereas cytoplasmic lncRNAs can act as competitive endogenous RNAs (ceRNAs) by sequestering miRNAs from sponging target mRNAs, thereby relieving the suppressive effects of miRNAs on mRNA expression [8, 12].

FLVCR1-AS1 is a tumor regulator involved in cell proliferation, migration, and invasion in numerous cancers, including lung, breast, and ovarian cancer [13–15]. Nevertheless, the biological functions and molecular mechanisms of FLVCR1-AS1 in PC remain unknown. Kruppel-like factor 10 (KLF10) is a DNA-binding transcriptional regulator that acts as a tumor suppressor by regulating the transcription level of target genes in multiple cancers, including PC and esophageal squamous cell carcinoma [16, 17]. However, whether KLF10 could function as a transcription factor to activate or repress non-coding RNAs remains unknown. Recent research has shown that KLF10 attenuates tumor progression in multiple cancers by modulating the PTEN/AKT pathway [18, 19].

Here, we investigated FLVCR1-AS1 expression levels in PC tumor tissues and para-nontumor tissues and found that FLVCR1-AS1 was significantly suppressed in PC tumor tissues. Moreover, analysis of clinicopathological data revealed that low FLVCR1-AS1 expression levels were associated with poor prognosis. Furthermore, functional studies demonstrated that FLVCR1-AS1 could act as a tumor suppressor by inhibiting PC cell proliferation and migration both *in vitro* and *in vivo*. We also revealed that FLVCR1-AS1 was directly transcribed by KLF10 and inversely upregulated KLF10 expression by sponging miR-513c-5p and miR-514b-5p and functioning as a ceRNA for KLF10 in PC. Thus, our results show the existence of a positive feedback loop between FLVCR1-AS1 and KLF10, which provides novel insights into PC tumorigenesis and a new therapeutic target for PC.

## Materials and methods

### Clinical samples

A total of 77 samples of human PC tissues and corresponding normal tissues were collected from patients admitted to the Ruijin Hospital affiliated with Shanghai Jiaotong University School of Medicine (Shanghai, China). All tissue samples were snap-frozen in liquid nitrogen and stored at -80 °C until use. The included patients did not receive any preoperative chemotherapy. The study protocols were approved by the institutional ethics committee of Ruijin Hospital.

### Cell culture and transfection

Human PC cell lines (Bxpc-3, CFPAC-1, MIA PaCa-2, PANC-1, and PATU-8988) and human normal pancreatic ductal epithelial cells (HPNE) were obtained from the Cell Bank of the Chinese Academy of Sciences. MIA PaCa-2, PANC-1, and PATU-8988 cells were cultured in DMEM, Bxpc-3 in RPMI-1640 medium, and CFPAC-1 in IMDM supplemented with 10 % fetal bovine serum (FBS).

For *in vitro* experiments, miR-513c-5p, miR-514b-5p, negative control (NC) mimics, KLF10 siRNA (si-KLF10), and NC siRNA (siRNA-NC) were synthesized by Bioegene (Shanghai, China). Full-length FLVCR1-AS1 and KLF10 cDNA were synthesized and inserted into a lentiviral vector or plasmid (Bioegene, Shanghai, China). PANC-1 and PATU-8988 cells transduced with lentivirus were treated with 2 µg/mL puromycin for 48 h to establish stable cell lines. All transfections were performed using Lipofectamine 3000. Cells were collected 48 h post-transfection. The miRNA mimics and siRNA sequences are listed in Tables S1-2.

### Quantitative real-time PCR (qRT-PCR) and subcellular fractionation

Total RNA was extracted from frozen pancreatic tissues and cell lines using TRIzol reagent (Invitrogen, Carlsbad, CA, USA). Reverse transcription was performed using the HiScript III RT SuperMix (Vazyme, Nanjing, China). Total RNA was detected using the AceQ Universal SYBR qPCR Master Mix (Vazyme, Nanjing, China). RNA from subcellular fractionation was separated using the PARIS Kit according to the manufacturer’s guidelines. ACTB and U6 expression levels were used as internal mRNA and miRNA controls, respectively. The primer sequences are listed in Table S3.

### Western blotting

Proteins were extracted from cells, boiled using RIPA buffer (Beyotime, Shanghai, China), loaded and separated on a 10 % SDS-PAGE gel (EpiZyme, Shanghai, China), transferred onto polyvinylidene fluoride membranes, and then incubated with the following primary antibodies: anti-KLF10, anti-CCND1, anti-CDK4, anti-CDK6, anti-E-cadherin, anti-N-cadherin, anti-vimentin, anti-PTEN, anti-p-AKT, anti-GAPDH, and anti-ACTB (Table S4). Protein expression was detected using the ECL reagent. ACTB and GAPDH were used as controls.

### Dual-luciferase reporter assay

A pGL3-based construct containing FLVCR1-AS1 and wild-type miRNA or corresponding mutant promoter sequences and the potential 3ʹ-UTR binding site of FLVCR1-AS1 and KLF10 or the matched mutant sequence were synthesized (Promega, Madison, WI, USA) and cloned into the reporter plasmids. Luciferase activity was measured using the dual-luciferase reporter assay system (Promega, USA) and normalized to Renilla luciferase activity.

### Mice model assay

Male BALB/c nude mice (4–6 weeks old) were purchased from the Chinese Academy of Sciences (Shanghai, China). Stably transfected PANC-1 and PATU-8988 cells were injected subcutaneously into the right flanks of six-week-old mice (5 × 10^6^ cells/0.2 mL PBS; *n* = 5, each group). The longest length (L) and width (W) were measured every four days from the first injection, and tumor volumes were measured as follows: volume = 1/2 (L × W^2^). Mice were sacrificed after 32 days, and the subcutaneous tumors were weighed, formalin-fixed, paraffin-embedded, and stained with hematoxylin–eosin (HE) or subjected to immunohistochemistry (IHC). For the *in vivo* tumor lung metastasis model, stably transfected PC cells (1 × 10^6^/0.1 mL PBS) were injected into the tail vein of each 4-week-old nude mouse (*n* = 5, each group). Six weeks after the cell injection, the mice were euthanized. For the *in vivo* tumor liver metastasis model, stably transfected PC cells (3 × 10^6^/50 µL PBS) were injected into the inferior hemi-spleen of 6-week-old nude mice (*n* = 5, each group). The mice were sacrificed six weeks after the cell injection. The lungs and livers were excised, formalin-fixed, paraffin-embedded, and stained with HE.

### RNA fluorescence *in situ* hybridization (FISH) and immunohistochemical (IHC) analysis

FISH was performed to detect the subcellular localization of FLVCR1-AS1 in PANC-1 and PATU-8988 cells, as previously described [20]. Cy3-labeled FLVCR1-AS1 probes were designed and synthesized by Servicebio (Wuhan, China). IHC analysis was performed using subcutaneous tumor tissues from xenograft tumor mice and PC tissues from patients, as previously described [20]. Ki-67, E-cadherin, vimentin, and KLF10 staining results were scored based on the proportion of positively stained cells [scored as 0 (0 %), 1 (1–25 %), 2 (26–50 %), 3 (51–75 %), or 4 (76–100 %)], and the intensity ranged from 0 to 3 (categorized as absent, weak, moderate, and strong). The histoscore was evaluated by multiplying the positive scores and intensity (total score range, 0–12).

### RNA immunoprecipitation (RIP)

RIP was performed using a Magna RNA-binding protein immunoprecipitation kit (Millipore, Bedford, MA, USA), according to the manufacturer’s instructions. Briefly, PANC-1 cells were collected and lysed in complete RIP lysis buffer containing magnetic beads conjugated with anti-AGO2 (Argonaute-2). The immunoprecipitated RNA was detected by qRT-PCR using normal rabbit IgG as the NC.

### Chromatin immunoprecipitation (ChIP)

The assay was performed using the SimpleChIP Plus Sonication Chromatin IP Kit (CST, USA), according to the manufacturer’s instructions. Briefly, PANC-1 cells were cross-linked with 1 % formaldehyde for 10 min and quenched with glycine. The nuclear lysates were sonicated to shear DNA fragments with lengths of 200–500 bp. The lysate was then immunoprecipitated with an anti-KLF10 or IgG antibody. The purified DNA fragments were detected using specific ChIP primers via qRT-PCR. The ChIP primers used are listed in Table S1.

### Flow cytometric analysis

To assess cell cycle arrest 48 h after transfection, PC cells were incubated in a 12-well plate. Two days later, the PC cells were fixed with 70 % ethanol and stored at 4 °C overnight. PC cells were harvested and resuspended in 400 µL of PI/RNase Staining Buffer (BD Biosciences, San Jose, CA, USA), stained for 15 min in a dark room at room temperature, and then assessed by flow cytometry.

### Cell proliferation assay

The CCK-8 (Cell count kit-8) assay (Meilunbio, Dalian, China) was used to determine cell proliferation. Transfected PC cells (2 × 10^3^) were collected at 48 h post-transfection and plated in a 96-well plate and incubated for another 5 days. Then, 10 µL of CCK-8 assay reagent was added at specific times and incubated for another 2 h, followed by measuring the absorbance at 450 nm. For the colony formation assay, 2 × 10^3^ cells/well were plated in 6-well plates and used to assess the proliferative ability of PC cells. After 2 weeks, the cells were fixed using 1 % crystal violet stain solution for 20 min at room temperature, and the number of colonies was counted manually. The EdU (5-ethynyl-20-deoxyuridine) labeling kit (Beyotime Biotechnology, Haimen, China) was used to determine cell viability. PC cells (2 × 10^4^) were plated in 24-well plates and cultured for 72 h. Afterwards, PC cells were incubated with EdU reagent for 2 h, fixed with 4 % paraformaldehyde and 0.5 % Triton X-100, followed by Hoechst staining. The EdU incorporation rate was counted manually to express the ratio of EdU-positive cells (green cells) to total Hoechst33342-positive cells (blue cells).

### Cell migration assays

Transfected PC cells (5 × 10^4^ cells/100 µL) were suspended in serum-free medium and plated in the top chambers. The lower chambers were filled with 700 µL of DMEM supplemented with 10 % FBS. After 24 h, the PC cells that migrated from the upper chamber were fixed with 1 % crystal violet stain solution for 20 min at room temperature and migrated cells were counted manually, and the relative cell migration percentage was calculated as the ratio of the number of migrated cells to that of the negative control. For the wound healing assays, after 48 h of transfection, wound areas were made using 200-µL pipette tips, and this time point was defined as 0 h. Afterwards, the cells were cultured for 36 h in serum-free medium after plating, and the wound areas were observed and photographed.

### Statistical analysis

Statistical analyses were performed using SPSS 20.0, and GraphPad Prism 7.0. Experiments were repeated independently at least three times and are presented as the mean ± standard deviation (SD). One-way ANOVA, Student’s t-test, and chi-square test were used to analyze the means between different groups. Survival curves were analyzed using the Kaplan–Meier method, and log-rank tests were used to evaluate differences between the groups. Statistical significance was set at *P* < 0.05.

## Results

### FLVCR1-AS1 expression is suppressed in PC tissues and cell lines and is correlated with grave prognosis and later pathological stage

**Fig. 1:**
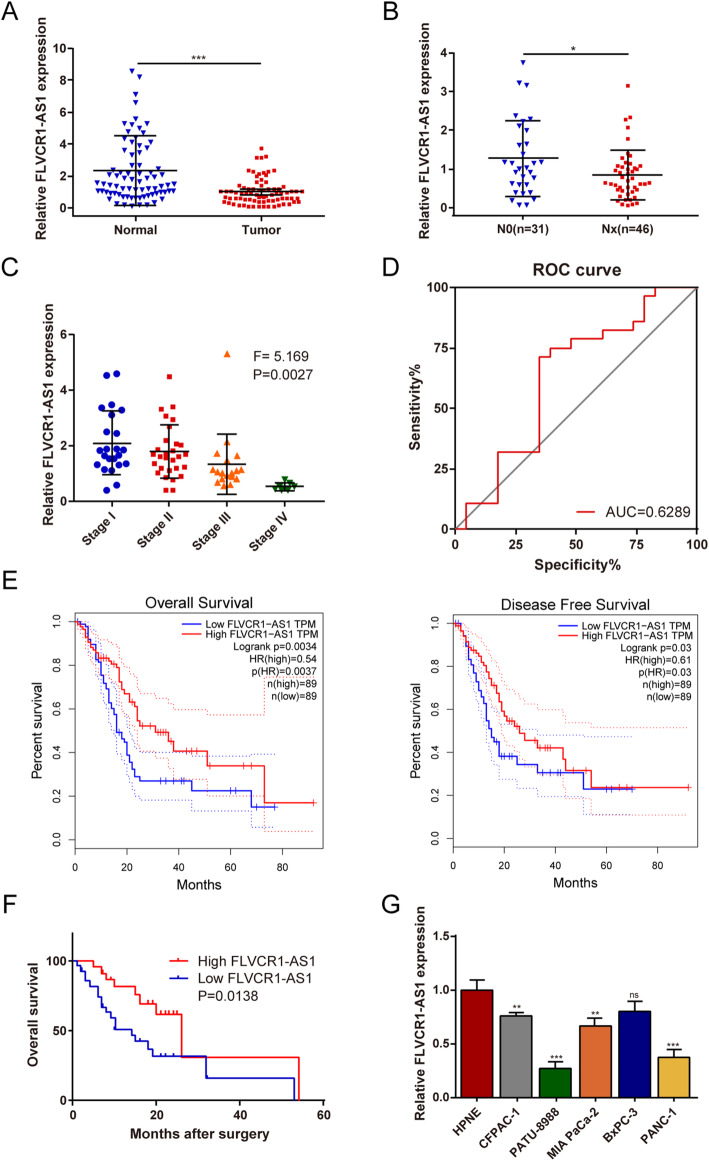
FLVCR1-AS1 expression is suppressed in PC tissues and cell lines and is correlated with later pathological stage and grave prognosis. **A** FLVCR1-AS1 expression in 77 pairs of PC tumor tissues and adjacent normal tissues. **B** The expression of FLVCR1-AS1 in PC patients with lymph node metastasis. **C** FLVCR1-AS1 expression in the included PC patients was divided according to cancer stage (*n* = 77). **D** By drawing the ROC curve, the demarcation points of low and high FLVCR1-AS1 expression were distinguished. **E** Overall survival (OS) and disease-free survival (DFS) based on FLVCR1-AS1 expression levels were determined using the Kaplan-Meier plotter databases. **F** Prognostic analysis of FLVCR1-AS1 using clinical prognostic data of 51 patients from our center. **G** FLVCR1-AS1 expression in PC cell lines (Bxpc-3, CFPAC-1, MIA PaCa-2, PANC-1, and PATU-8988) compared with that of normal pancreatic ductal epithelial cell line HPNE detected by qRT-PCR. *P < 0.05; ***P < 0.001. All experiments were repeated three times

Analysis of 77 paired postoperative PC and noncancerous tissues revealed that FLVCR1-AS1 expression was significantly inhibited in PC tissues compared to that in non-tumor tissues (Fig. 1A). To further explore the clinicopathological and prognostic significance of FLVCR1-AS1 in PC, clinical data were integrated and analyzed. We found that FLVCR1-AS1 was notably suppressed in PC patients and was inversely correlated with lymph node metastasis (Fig. 1B) and advanced pathological stage (Fig. 1C). By drawing the ROC curve, the demarcation points of low and high FLVCR1-AS1 expression were distinguished (Fig. 1D). Notably, we found that high FLVCR1-AS1 expression levels were correlated with better prognosis using clinical data from both the TCGA database and our center (Fig. 1E-F). Subsequently, we evaluated the correlations between FLVCR1-AS1 expression and clinical characteristics and found that FLVCR1-AS1 expression was significantly correlated with pathological stage (*P* = 0.001) and lymph node metastasis (*P* = 0.007) (Table 1). Moreover, univariate and multivariate analyses demonstrated that FLVCR1-AS1 expression was an independent prognostic indicator for PC patients with significant hazard ratios (HR, 0.431; 95 % CI 0.178–0.715, *P* = 0.028). Additionally, FLVCR1-AS1 mRNA expression was downregulated in four PC cell lines (CFPAC-1, MIA PaCa-2, PANC-1, and PATU-8988) compared to that in the human pancreatic epithelial cell line HPNE (Fig. 1G). Collectively, these results suggest that FLVCR1-AS1 is significantly suppressed in PC and that FLVCR1-AS1 overexpression is associated with a better prognosis.

**Table 1:** Correlations between FLVCR1-AS1 expression and clinical characteristics in PC patients

Clinicopathologic parameters	Case (*n* = 77)	FLVCR1-AS1 expression		*P value*
		Low	High	
Total	77	46	31	
Gender				0.611
Male	42	24	18	
Female	35	22	13	
Age				0.89
≥ 60	39	23	16	
< 60	38	23	15	
Pathological stage				0.001*
I	23	9	14	
II	29	15	14	
III-IV	25	22	3	
T stage				0.329
T1-2	35	23	12	
T3-4	42	23	19	
Lymph node metastasis				0.007*
N0	31	20	11	
N1	34	15	19	
N2	12	11	1	
Distant metastasis				0.061
M0	70	39	31	
M1	7	7	0	

### FLVCR1-AS1 inhibits PC cell proliferation and migration *in vitro*

**Fig. 2:**
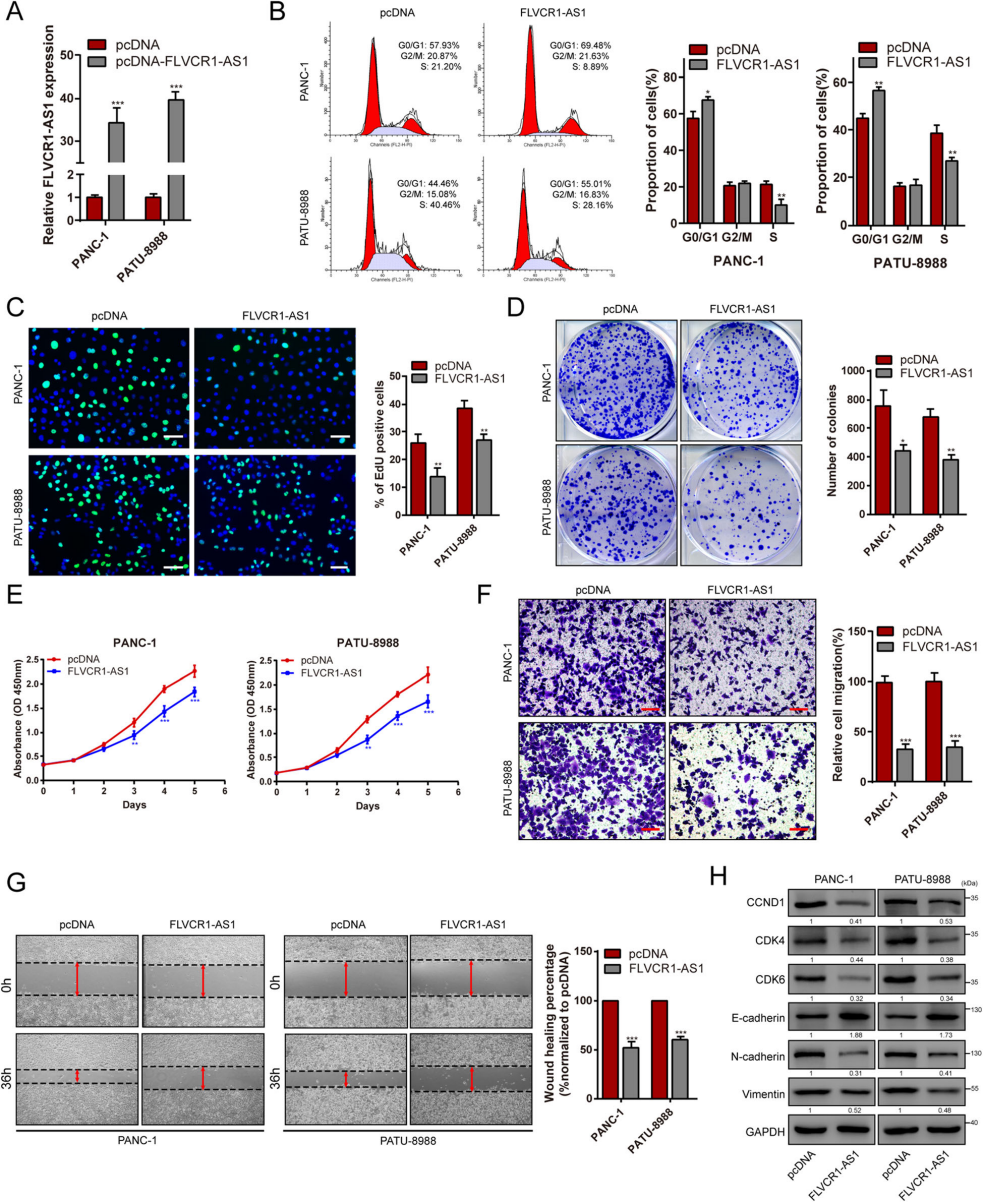
FLVCR1-AS1 inhibited proliferation, cell cycle, and migration of PC cells *in vitro*. **A** FLVCR1-AS1 overexpression was verified by qRT-PCR. **B** Cell cycle arrest was determined by flow cytometry to detect the proportion of proliferating PC cells after FLVCR1-AS1 overexpression. **C** The EdU assay was performed to determine the cell proliferative potential of FLVCR1-AS1 overexpressing PC cells. **D** PANC-1 and PATU-8988 cells were cultured in 6-well plates after transfection with pcDNA-FLVCR1-AS1. **E** PANC-1 and PATU-8988 cells were subjected to CCK-8 assays after transfection with pcDNA-FLVCR1-AS1. **F** Transwell migration assay of PANC-1 and PATU-8988 cells after FLVCR1-AS1 overexpression. **G** Wound healing assays performed using PANC-1 and PATU-8988 cells after FLVCR1-AS1 overexpression. **H** Expression of cell cycle-related proteins and metastasis-related proteins was evaluated by western blotting after FLVCR1-AS1 overexpression. Scale bar = 50 μm. *P < 0.05, **P < 0.01, and ***P < 0.001. All experiments were repeated three times

To elucidate the role of FLVCR1-AS1 in PC, PANC-1 and PATU-8988 cells were transfected with pcDNA-FLVCR1-AS1. FLVCR1-AS1 overexpression was verified using qRT-PCR (Fig. 2A). First, the cell cycle progression of FLVCR1-AS1 overexpressing PC cells was determined by flow cytometry. We found that FLVCR1-AS1 overexpression induced cell cycle arrest (Fig. 2B). Next, EdU, colony formation, and CCK-8 assays were used to determine the effect of FLVCR1-AS1 on PC cell proliferation. We found that FLVCR1-AS1 overexpression inhibited PC cell proliferation (Fig. 2C-E). Subsequently, Transwell and wound healing assays were used to determine the effects of FLVCR1-AS1 on PC cell migration. Transwell assays suggested that FLVCR1-AS1 overexpression inhibited the migration ability of PANC-1 and PATU-8988 cells (Fig. 2F). Additionally, we found that pcDNA-FLVCR1-AS1-transfected PC cells showed slower wound healing than pcDNA-transfected PC cells (Fig. 2G). Western blotting indicated that the cell cycle-related proteins CCND1, CDK4, and CDK6 were inhibited after transfection with pcDNA-FLVCR1-AS1. In addition, the epithelial marker E-cadherin was significantly upregulated in FLVCR1-AS1 overexpressing PC cells. Conversely, the protein levels of mesenchymal markers, such as N-cadherin and vimentin, were decreased (Fig. 2H). Altogether, these results suggest that FLVCR1-AS1 can affect tumor progression by inhibiting PC cell proliferation and migration.

### FLVCR1-AS1 inhibits PC cell proliferation and metastasis *in vivo*

**Fig. 3:**
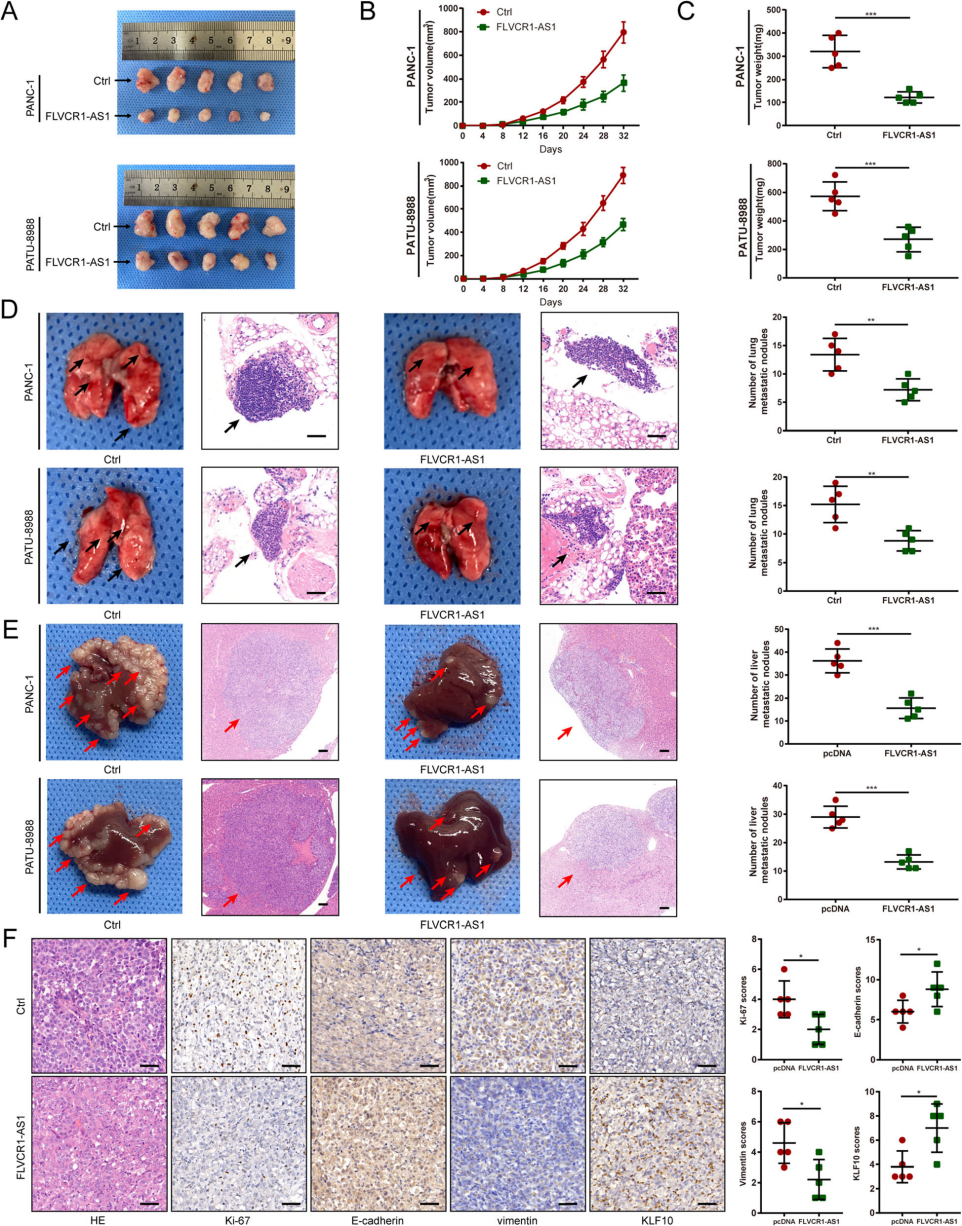
FLVCR1-AS1 inhibits PC cell growth and metastasis *in vivo*. **A** Images of subcutaneous tumors. **B** The tumor volume was calculated every 4 days after injection. **C** The tumor weights of the subcutaneous xenografts. **D** Representative photographs of the lung tissues and HE staining of lung metastatic nodules with original magnification: ×40. Scale bar = 100 μm. **E** Representative photographs of the liver tissues and HE staining of liver metastatic nodules with original magnification: ×10. Scale bar = 100 μm. **F** Representative photographs of HE, Ki-67, E-cadherin, vimentin, and KLF10 IHC staining in xenograft tumors. Scale bar = 50 μm. *P < 0.05, **P < 0.01, and ***P < 0.001

To explore the biological functions of FLVCR1-AS1 *in vivo*, PANC-1 and PATU-8988 cells stably overexpressing FLVCR1-AS1 or an empty vector were subcutaneously or tail intravenously injected into nude mice (*n* = 5 per group). We found that xenograft tumor volumes in the FLVCR1-AS1 group were smaller than those in the control group (Fig. 3A). Tumor volume was measured every 4 days after injection, and we found that the FLVCR1-AS1 group had slower tumor growth and tumors with lower weights than the control group (Fig. 3B-C). For lung metastasis model, the number of lung metastatic nodules in the FLVCR1-AS1 group was lower than that in the control group. Moreover, for liver metastasis model, the number of liver metastatic nodules in the FLVCR1-AS1 group was also lower than that in the control group. (Fig. 3D-E). To validate the ability of FLVCR1-AS1 to mediate tumor growth and metastasis, subcutaneous tumor tissues of PATU-8988 groups were stained with antibodies against HE, Ki-67, E-cadherin, and vimentin for IHC. The expression of Ki-67, a proliferation marker, was decreased in FLVCR1-AS1-overexpressing PC tumors, while the expression of E-cadherin, an epithelial marker, was increased. Meanwhile, the expression of vimentin, a mesenchymal marker, was suppressed in FLVCR1-AS1 overexpressing PC tumors. Notably, these results were consistent with the *in vitro* findings that FLVCR1-AS1 overexpression inhibited PC cell tumor growth and metastasis (Fig. 3F).

### FLVCR1-AS1 functions as a miR-513c-5p and miR-514b-5p sponge in PC

**Fig. 4:**
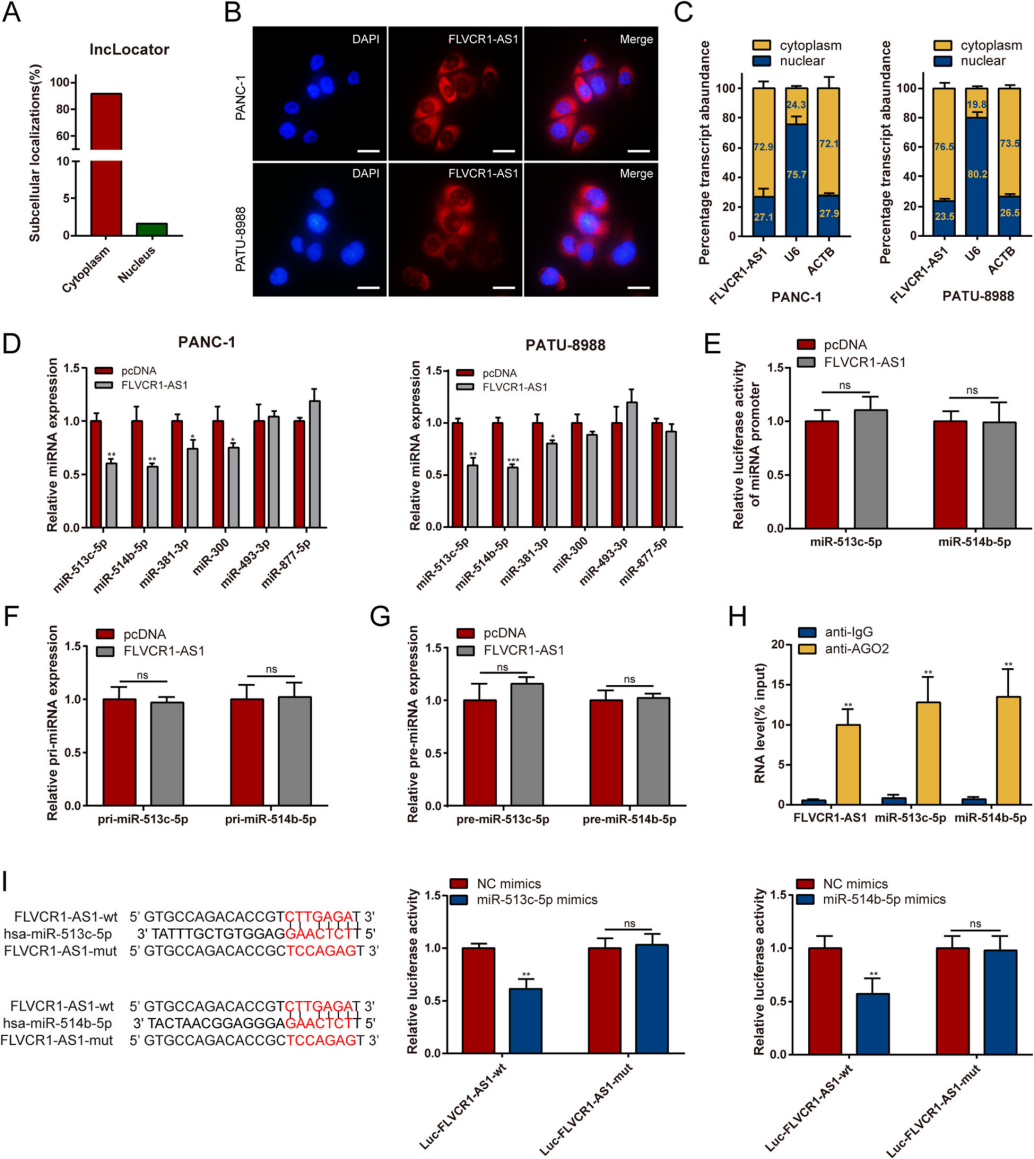
FLVCR1-AS1 functions as a miR-513c-5p and miR-514b-5p sponge in PC. **A** Relative FLVCR1-AS1 expression in subcellular localization predicted by lncLocator. **B** Representative FISH images showing the expression of FLVCR1-AS1 in PANC-1 and PATU-8988 cells (RED). The nucleus was stained using DAPI (BLUE). **C** Relative FLVCR1-AS1 expression levels in subcellular fractions. **D** The expression of six potential target miRNAs in PANC-1 and PATU-8988 cells after FLVCR1-AS1 overexpression. **E** Promoter luciferase activity of miR-513c-5p and miR-514b-5p in 293-T cells overexpressing FLVCR1-AS1. **F** The expression of pri-miR-513c-5p and pri-miR-514b-5p in PANC-1 cells transfected with empty control or pcDNA-FLVCR1-AS1. **G** The expression of pre-miR-513c-5p and pre-miR-514b-5p in PANC-1 cells transfected with empty control or pcDNA-FLVCR1-AS1. **H** RIP assay was performed using rabbit AGO2 and IgG antibodies in PANC-1 cells. Relative expression levels of FLVCR1-AS1, miR-513c-5p, and miR-514b-5p were determined by qRT-PCR. **I** Luciferase activity in 293-T cells co-transfected with FLVCR1-AS1 wild-type or mutant sequence and miR-513c-5p or miR-514b-5p mimics. Scale bar = 20 μm. **P < 0.01; ns, no significance. All experiments were repeated three times

To investigate the underlying mechanism of FLVCR1-AS1-induced tumor suppression, we first verified its subcellular localization, as the function of lncRNAs depends on their subcellular distribution [21]. The online tool lncLocator (http://www.csbio.sjtu.edu.cn/bioinf/lncLocator/) was used to predict the subcellular localization of FLVCR1-AS1. We found that FLVCR1-AS1 was mainly expressed in the cytoplasm (Fig. 4A), which was verified by FISH and subcellular fractionation assays (Fig. 4B-C). Because many cytoplasmic lncRNAs function as ceRNAs by binding to miRNAs, we hypothesized that FLVCR1-AS1 could bind to miRNAs in PC. Six potential FLVCR1-AS1 target miRNAs (miR-513c-5p, mir-514b-5p, miR-381-3p, miR-300, miR-493-3p, and miR-877-5p) were predicted using starBase 3.0 (http://starbase.sysu.edu.cn/) and their mRNA expression after FLVCR1-AS1 overexpression was verified by qRT-PCR. Among the six candidates, miR-513c-5p and miR-514b-5p were shown to be significantly downregulated in both FLVCR1-AS1-overexpressing PANC-1 and PATU-8988 cells (Fig. 4D). To exclude the possibility that FLVCR1-AS1 regulates miRNA transcription, pri-miRNA, or pre-miRNA synthesis, we assessed the effect of FLVCR1-AS1 in the promoter regions and miR-513c-5p and miR-514b-5p pri-miRNA or pre-miRNA expression and determined that FLVCR1-AS1 did not affect promoter activity or pri-miRNA and pre-miRNA expression (Fig. 4E-G).

Additionally, the RIP results revealed that FLVCR1-AS1, miR-513c-5p, and miR-514b-5p were all significantly enriched in AGO2-containing microribonucleoprotein complexes, indicating that AGO2 could directly bind to FLVCR1-AS1, miR-513c-5p, and miR-514b-5p in PANC-1 cells (Fig. 4H). Dual-luciferase reporter assays were used to determine whether miRNAs could directly interact with FLVCR1-AS1 in 293T cells. The predicted binding site of FLVCR1-AS1 was mutated compared to that of NC mimics. Transfection with miR-513c-5p or miR-514b-5p mimics and Luc-FLVCR1-AS1-wt, but not Luc-FLVCR1-AS1-mut, led to a significant decrease in fluorescence in 293T cells (Fig. 4I). This indicates that FLVCR1-AS1 may interact with miR-513c-5p and miR-514b-5p, thus acting as a ceRNA and molecular sponge for miR-513c-5p and miR-514b-5p in PC cells.

### miR-513c-5p and miR-514b-5p are upregulated in PC with poor prognosis and participate in the anti-tumor effects of FLVCR1-AS1 in PC cells

**Fig. 5:**
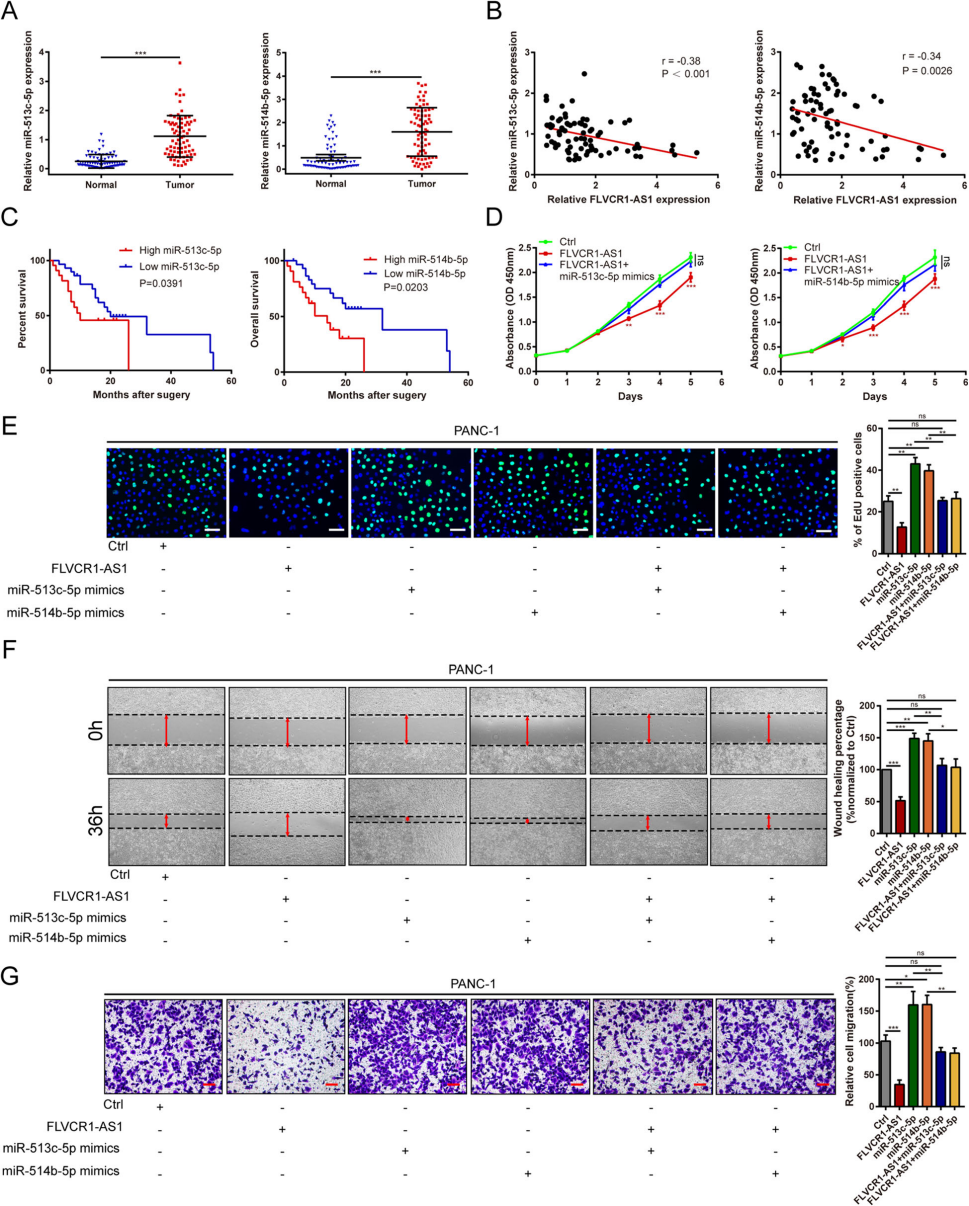
miR-513c-5p and miR-514b-5p are upregulated in PC and are associated with poor prognosis and promote proliferation and migration in PC cells. **A** miR-513c-5p and miR-514b-5p expression in 77 PC tissues compared to matched normal tissues. **B** The correlation between FLVCR1-AS1 and miR-513c-5p and miR-514b-5p expression in 77 PC tissue samples. **C** Prognostic analysis of miR-513c-5p and miR-514b-5p using survival data of 51 patients from our center. **D** CCK-8 assays showed that FLVCR1-AS1 overexpression inhibits PANC-1 cell proliferation. Co-transfection of miR-513c-5p or miR-514b-5p mimics and pcDNA-FLVCR1-AS1 eliminated the decrease in proliferation induced by FLVCR1-AS1 overexpression. **E** EdU assays showed that FLVCR1-AS1 overexpression inhibits PANC-1 cell proliferation. miR-513c-5p or miR-514b-5p overexpression promotes PANC-1 cell proliferation. Co-transfection of miR-513c-5p or miR-514b-5p mimics with pcDNA-FLVCR1-AS1 eliminated the decrease in proliferation rates. **F**-**G** Wound healing and Transwell assays showed that FLVCR1-AS1 overexpression decreases PANC-1 cell migration, while miR-513c-5p or miR-514b-5p overexpression promotes PANC-1 cell migration. Co-transfection of miR-513c-5p or miR-514b-5p mimics with pcDNA-FLVCR1-AS1 abolished the decrease in migration ability in PANC-1 cells. Scale bar = 50 μm *P < 0.05; **P < 0.01; ***P < 0.001; ns, no significance. All experiments were repeated three times

The specific functions and underlying mechanisms of miR-513c-5p and miR-514b-5p in PC remain unknown. Thus, miR-513c-5p and miR-514b-5p expression levels in 77 matched PC and non-tumor tissues were detected. miR-513c-5p and miR-514b-5p were highly expressed in PC tissues compared to non-tumor tissues (Fig. 5A). Correlation analysis revealed a negative correlation between FLVCR1-AS1 and miR-513c-5p or miR-514b-5p in PC tissues (Fig. 5B). High miR-513c-5p and miR-514b-5p expression levels were correlated with worse prognosis using clinicopathological data from TCGA (Fig. S1) and our center (Fig. 5C). Next, the CCK-8 and EdU assays were used to investigate whether the antiproliferative effects of FLVCR1-AS1 were dependent on miR-513c-5p and miR-514b-5p sponging. The CCK-8 assay results demonstrated that miR-513c-5p or miR-514b-5p upregulation could rescue FLVCR1-AS1-induced growth inhibition of PANC-1 cells (Fig. 5D). Moreover, EdU assays showed that FLVCR1-AS1 overexpression significantly inhibited PANC-1 cell proliferation compared to controls, which could be rescued by transfection with miR-513c-5p or miR-514b-5p mimics (Fig. 5E). Transwell and wound healing assays were performed to evaluate whether miR-513c-5p or miR-514b-5p participated in FLVCR1-AS1-induced migration inhibition in PANC-1 cells. As expected, our results confirmed that FLVCR1-AS1 overexpression inhibited the migration of PANC-1 cells, while transfection with miR-513c-5p or miR-514b-5p mimics rescued the migration inhibition of PANC-1 cells (Fig. 5F). Similarly, Transwell assays suggested that FLVCR1-AS1 overexpression significantly suppressed the migration ability of PANC-1 cells, and this effect could be partially eliminated after transfection with miR-513c-5p or miR-514b-5p mimics (Fig. 5G). Collectively, these results suggest that FLVCR1-AS1 inhibits PC progression by functioning as a ceRNA for miR-513c-5p and miR-514b-5p.

### FLVCR1-AS1 serves as a ceRNA to modulate KLF10 expression in PC

**Fig. 6:**
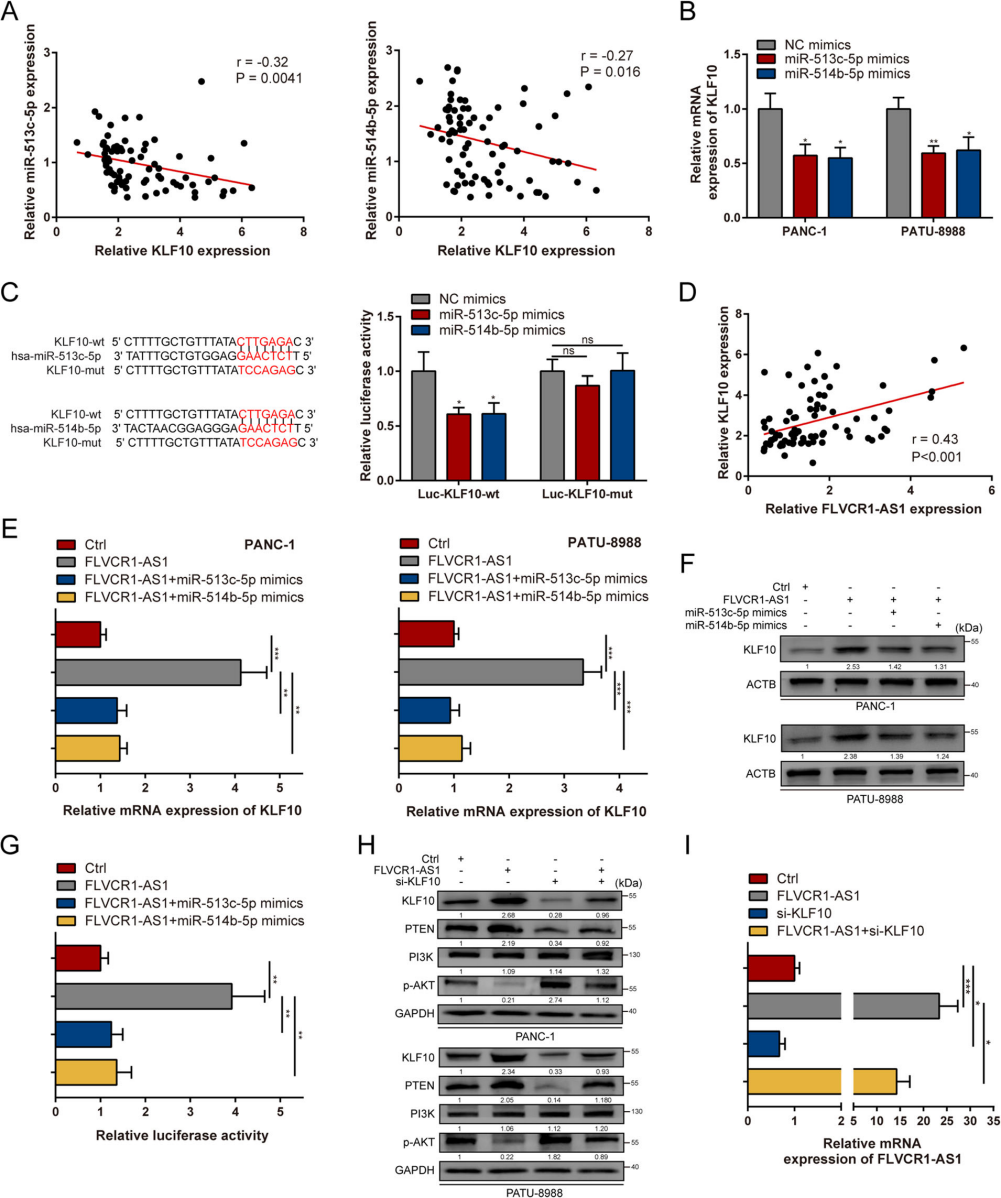
FLVCR1-AS1 serves as a ceRNA to modulate KLF10 expression in PC. **A** The correlation between KLF10 and miR-513c-5p and miR-514b-5p expression in 77 PC tissue samples. **B** KLF10 expression in PANC-1 and PATU-8988 cells after transfection with miR-513c-5p or miR-514b-5p mimics. **C** Luciferase activity in 293T cells co-transfected with KLF10 wild-type or mutant sequence and miR-513c-5p or miR-514b-5p mimics. **D** The correlation between FLVCR1-AS1 and KLF10 expression levels in 77 PC tissue samples. **E**-**F** KLF10 expression was evaluated by qRT-PCR and western blotting in PANC-1 and PATU-8988 cells transfected with pcDNA-FLVCR1-AS1, miR-513c-5p, and miR-514b-5p mimics. **G** Luciferase activity of Luc-KLF10 with indicated treatment in 293T cells. **H** KLF10, PTEN, and p-AKT protein levels were detected by western blotting in PC cells with indicated treatment. **I** FLVCR1-AS1 mRNA expression was evaluated by qRT-PCR in PANC-1 cells with indicated treatment. *P < 0.05; **P < 0.01; ***P < 0.001; ns, no significance. All experiments were repeated three times

The specific functions of miRNAs depend on their downstream targets. Thus, the potential targets of miR-513c-5p and miR-514b-5p were investigated. Using the RAID, miRDB, miRTarBase, and TargetScan databases, KLF10 was identified as a potential target of both miR-513c-5p and miR-514b-5p (Fig. S2). To investigate whether miR-513c-5p or miR-514b-5p could sponge KLF10 directly, we determined their expression levels in 77 PC tissue samples and found that KLF10 expression levels were negatively correlated with miR-513c-5p or miR-514b-5p expression (Fig. 6A). Additionally, KLF10 expression was suppressed in PC tissues compared to that in normal tissues (Fig. S3A), and low KLF10 expression levels were associated with a poor prognosis (Fig. S3B). IHC assays were then used to determine KLF10 protein levels in 25 PC tissues and paired non-cancerous tissues, revealing that KLF10 protein levels were lower in PC tissues than in paired non-tumor tissues (Fig. S3C). Moreover, IHC assays showed that KLF10 expression levels were significantly increased in xenografts formed from FLVCR1-AS1 overexpressing cells (Fig. 3E). KLF10 protein levels were also lower in PC tissues with low FLVCR1-AS1 expression (Fig. S3D). Additionally, KLF10 mRNA expression levels decreased after transfection with miR-513c-5p or miR-514b-5p mimics in both PANC-1 and PATU-8988 cells (Fig. 6B). Dual-luciferase reporter assays showed that transfection with miR-513c-5p or miR-514b-5p mimics decreased the luciferase activity of the Luc-KLF10-wt but not the Luc-KLF10-mut group (Fig. 6C). Furthermore, we found that KLF10 expression levels were positively correlated with FLVCR1-AS1 expression levels in PC (Fig. 6D). In both PANC-1 and PATU-8988 cells, increased FLVCR1-AS1 expression levels significantly upregulated KLF10 mRNA and protein levels. Moreover, transfection with miR-513c-5p or miR-514b-5p mimics eliminated the increase in KLF10 expression levels in FLVCR1-AS1 overexpressing PC cells (Fig. 6E-F). Additionally, transfection with FLVCR1-AS1 increased Luc-KLF10-wt luciferase activity, and co-transfection with FLVCR1-AS1 and miR-513c-5p or miR-514b-5p mimics abolished this effect (Fig. 6G). Western blotting indicated that FLVCR1-AS1 significantly repressed AKT phosphorylation by upregulating PTEN, which could be abolished by silencing KLF10, while PI3K was not regulated by FLVCR1-AS1 (Fig. 6H). Collectively, these results suggest that FLVCR1-AS1 can release KLF10 by sequestering endogenous miR-513c-5p and miR-514b-5p, thus modulating PC cell progression via the PTEN/AKT pathway.

### The tumor suppression effects of FLVCR1-AS1 depend on KLF10

To explore whether KLF10 is involved in the FLVCR1-AS1-induced tumor inhibitory effects in PC, we first verified the effect of KLF10 on FLVCR1-AS1-induced inhibition of cell proliferation. The CCK-8 assay results revealed that silencing of KLF10 partially eliminated FLVCR1-AS1 overexpression-induced suppression of PANC-1 cell proliferation (Fig. S3F). Likewise, the EdU assay results demonstrated that transfection with si-KLF10 abolished FLVCR1-AS1 overexpression-induced growth inhibition in PANC1 cells (Fig. S3G). Western blotting indicated that FLVCR1-AS1 overexpression-induced inhibition of cell cycle-related proteins CCND1, CDK4, and CDK6 could be reversed after transfection with si-KLF10 (Fig. S3E). Subsequently, wound healing and transwell assays were performed to determine the effects of KLF10 knockdown on FLVCR1-AS1-induced migration inhibition in PANC-1 cells. We found that FLVCR1-AS1 overexpression-induced migration inhibition could be rescued by KLF10 silencing in PANC-1 cells (Fig. S3G-H). Additionally, western blotting showed that FLVCR1-AS1-induced E-cadherin protein upregulation and N-cadherin and vimentin protein downregulation could be impaired by KLF10 knockdown (Fig. S3E). We also performed qRT-PCR to detect FLVCR1-AS1 mRNA expression in PANC-1 cells transfected with si-KLF10 and found that silencing of KLF10 suppressed FLVCR1-AS1 expression (Fig. 6I). Together, these data suggest that FLVCR1-AS1 suppresses PC tumorigenesis and development by modulating KLF10.

### FLVCR1-AS1 is a direct transcriptional target of KLF10

**Fig. 7:**
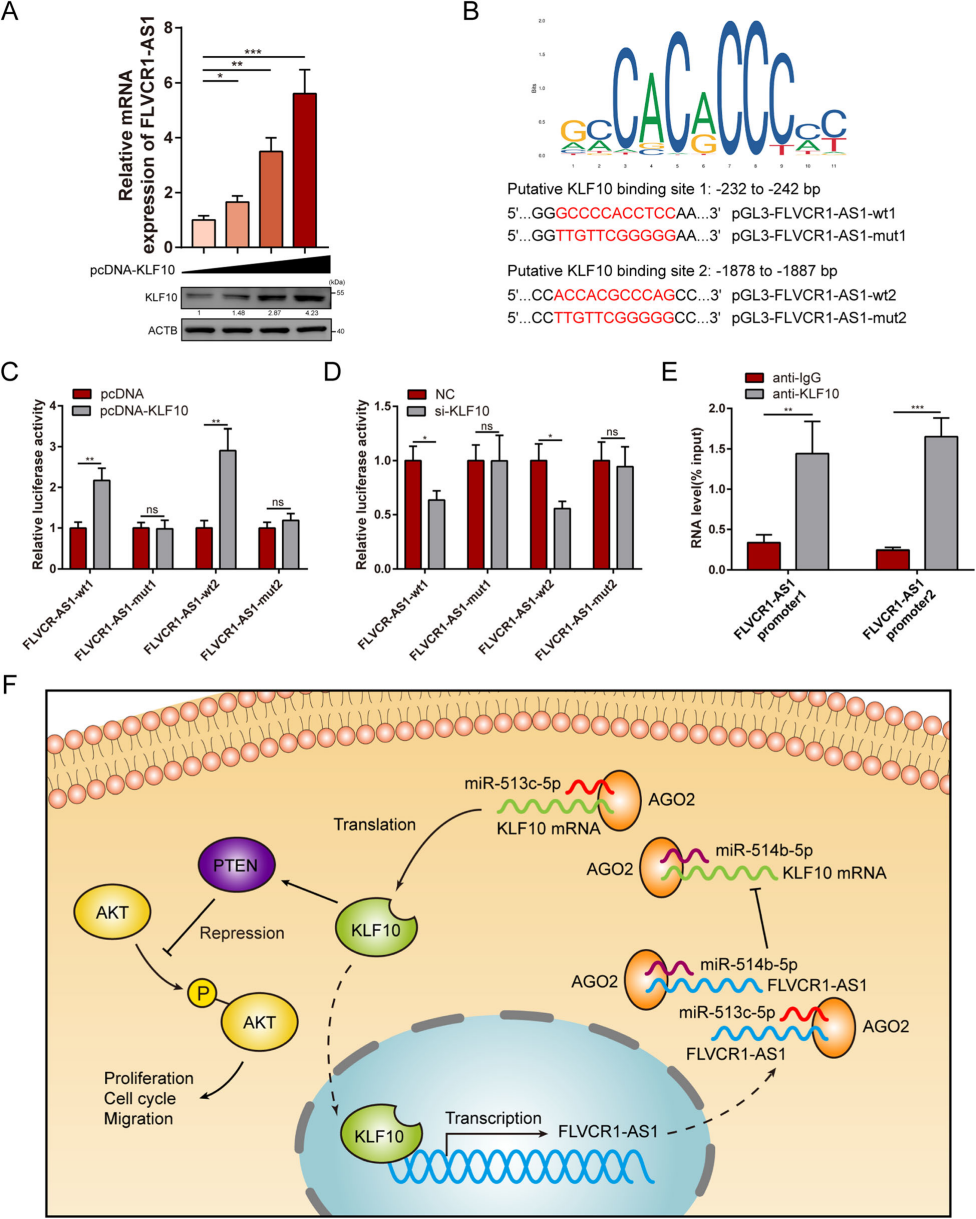
FLVCR1-AS1 is a direct transcriptional target of KLF10. **A** PANC-1 cells were transfected with 0.5, 1, or 2 µg of pcDNA-KLF10. PATU-8988 cells were transfected with si-KLF10 and negative control. At 48 h post-transfection, KLF10 protein levels and FLVCR1-AS1 mRNA levels were detected by Western blotting and qRT-PCR, respectively. **B** The upper picture represents the KLF10 binding motif provided by the JASPAR database. Dual-luciferase reporters were constructed with either of the two putative KLF10 binding sites and corresponding mutant binding sites in the FLVCR1-AS1 gene promoter. **C**-**D** Luciferase activity in 293T cells co-transfected with indicated luciferase reporter plasmids and pcDNA-KLF10 or si-KLF10. **E** ChIP assays were used to evaluated KLF10 binding to the FLVCR1-AS1 promoter region. **F** Proposed model demonstrating a positive feedback loop between FLVCR1-AS1 and KLF10 in inhibiting proliferation and migration in PC. *P < 0.05; **P < 0.01; ***P < 0.001; ns, no significance. All experiments were repeated three times

KLF10 is thought to function as an anti-oncogene transcription factor. Thus, we hypothesized that KLF10 may modulate FLVCR1-AS1 at the transcriptional level. Consequently, we found that KLF10 overexpression significantly increased FLVCR1-AS1 expression levels in a dose-dependent manner (Fig. 7A). By using promoter sequence analysis tools (UCSC and JASPAR), two potential KLF10 binding sites (GCCCCACCTCC/ACCACGCCCAG) were detected in the promoter region of the FLVCR1-AS1 gene (Fig. 7B). Next, we investigated whether KLF10 could directly interact with the two putative promoter binding sites in 293-T cells using a dual-luciferase reporter assay. As expected, the results revealed that luciferase activity was increased in the reporter containing two individual wild-type binding sites, while the reporter containing mutant sites did not respond to either KLF10 overexpression or knockdown (Fig. 7C-D). In addition, ChIP assays performed on the two predicted binding sites in the FLVCR1-AS1 promoter region revealed that KLF10 could directly bind to the FLVCR1-AS1 promoter region and partly activate FLVCR1-AS1 transcription (Fig. 7E).

## Discussion

PC is one of the most fatal digestive tract malignancies and is characterized by late diagnosis, rapid tumor progression, and grave prognosis. Although several comprehensive clinical trials using gemcitabine-based chemotherapeutics have been conducted on patients with PC, they have shown few benefits [1, 22]. Thus, it is essential to elucidate the molecular mechanismsmechanism of PC tumorigenesis and identify new therapeutic targets to improve prognosis. Recently, lncRNAs have been shown to play essential roles in multiple cancers, namely in tumorigenesis, tumor growth, metastasis, angiogenesis, tumor cell stemness, and drug resistance [23, 24]. Moreover, aberrantly expressed lncRNAs have been shown to play crucial functions in PC. For instance, lncRNA PVT1 triggers cytoprotective autophagy and promotes PC progression by binding to miR-20a-5p [25], while the lncRNA LINC00261 inhibits c-Myc-mediated aerobic glycolysis in PC by sponging miR-222-3p and sequestering IGF2BP1 [26]. This indicates that investigating the role of lncRNAs in PC could contribute to the discovery of novel therapeutic targets and strategies. The lncRNA FLVCR1-AS1 was initially reported to act as an oncogene in hepatocellular carcinoma (HCC) by promoting proliferation, migration, and invasion, as well as inhibiting apoptosis [27]. Similar results have been reported for lung cancer, gastric cancer, and cholangiocarcinoma [28–30]. However, the role of FLVCR1-AS1 in PC remains unclear. Thus, FLVCR1-AS1 expression levels in PC were analyzed and we found that FLVCR1-AS1 levels were significantly decreased in PC tissues and cells, which was associated with a poor prognosis. The results of our functional studies demonstrated that FLVCR1-AS1 overexpression could inhibit PC cell proliferation and metastasis both *in vitro* and *in vivo*, indicating a tumor-suppressive role in PC.

Regarding the underlying mechanism, evidence suggests that lncRNA functions are determined by their subcellular localization [11]. Therefore, nucleus-localized lncRNAs may be involved in transcriptional regulation, while cytoplasm-localized lncRNAs may act as ceRNAs by sponging miRNAs [8, 12]. Here, we found that FLVCR1-AS1 was mainly enriched in the cytoplasm of PC cells, implying that it may act as a ceRNA by binding to miRNAs. Subsequently, we determined that FLVCR1-AS1 contains binding sites for miR-513c-5p and miR-514b-5p via bioinformatics analysis and found that FLVCR1-AS1 overexpression significantly decreased miR-513c-5p and miR-514b-5p expression levels. Additionally, we confirmed that FLVCR1-AS1 directly interacts with miR-513c-5p or miR-514b-5p via dual-luciferase reporter assays and RIP assays.

MiR-513c-5p was shown to act as a tumor regulator in various cancers, such as glioblastoma, hepatocellular carcinoma, and neuroblastoma, by modulating cell viability, cell cycle, invasion, migration, and apoptosis [31–33]. Moreover, miR-514b-5p expression was shown to be significantly elevated in colorectal cancer, which may play a role in accelerating tumor metastasis [34]. However, the role of miR-513c-5p or miR-514b-5p in PC remains unknown. Our results demonstrated that miR-513c-5p or miR-514b-5p expression levels are significantly elevated in PC and are associated with poor prognosis. Additionally, an inverse correlation between FLVCR1-AS1 and miR-513c-5p or miR-514b-5p expression was observed in PC tissues. Notably, transfection with miR-513c-5p or miR-514b-5p mimics partially reversed FLVCR1-AS1 overexpression-mediated inhibition of PC cell proliferation and migration. Collectively, our results indicated that FLVCR1-AS1 could function as a ceRNA by binding to miR-513c-5p and miR-514b-5p in PC.

KLF10 functions as an anti-oncogene transcription factor via TGF-β signaling in multiple cancers by playing a role in apoptosis, cell cycle regulation, cell growth, and differentiation [35]. Additionally, a variety of protein-coding genes have recently been shown to be downstream targets of KLF10. For example, KLF10 can directly bind to the FOXP3 core promoter and act as a switch to alternatively activate or repress FOXP3 transcription by interacting with the HAT or HDAC pathways [36]. KLF10 also directly binds to stathmin and suppresses its promoter activity, thus inhibiting proliferation and promoting apoptosis in TGF-β-susceptible HCC cells [37]. However, little research has been conducted on KLF10-mediated regulation of non-coding RNA promoter activity. Here, we first established that KLF10 could increase lncRNA FLVCR1-AS1 expression by activating FLVCR1-AS1 transcription, which is involved in PC tumorigenesis and development. Additionally, we found that FLVCR1-AS1 functions as a ceRNA to sequester miR-513c-5p or miR-514b-5p from sponging KLF10 mRNA, relieving their suppressive effects on KLF10 expression and consequently inhibiting the PTEN/AKT pathway.

Our study has some limitations. We investigated the tumor suppressor function of FLVCR1-AS1 in the cytoplasm. However, the regulatory mechanism of FLVCR1-AS1 in the nucleus requires further study. Our study reported the transcription-promoting regulatory effect of KLF10 on FLVCR1-AS1 and the positive feedback pathway between FLVCR1-AS1 and KLF10; however, the underlying mechanism leading to FLVCR1-AS1 downregulation in PC remains unknown. In addition, in previous studies on the role of FLVCR1-AS1 in other human cancers, FLVCR1-AS1 mainly acted as an oncogene. To further explain this phenomenon, we propose several hypotheses. First, the discrepancy between these results may be due to tissue specificity in different tumors. PC cells are surrounded by a large number of mesenchymal cells and their secreted intercellular matrix; thus, compared with other human cancers, a special tumor microenvironment is the most significant pathological feature of PC. These mesenchymal cells not only protected PC cells from chemotherapeutic drugs and immune surveillance, but also masked many of the PC cell signals by secreting multitudinous intercellular factors, which may lead to specific tumor characteristics. To adapt to the complex tumor microenvironment, the functions of several genes in PC may exacerbate the tumorigenesis of PC. Second, in our study, we mainly investigated the cytoplasm-localized FLVCR1-AS1 by performing lncLocator, FISH, and subcellular fractionation assays. However, other previous cancer studies did not clarify the localization of FLVCR1-AS1 by experimental validation in detail [15, 27, 30]. FLVCR1-AS1 localizes to different subcellular distributions in cancers other than PC, and may perform different functions in these different localizations. Third, FLVCR1-AS1 was upregulated in other cancers and may facilitate tumorigenesis signaling mechanically, while overexpressed FLVCR1-AS1 could activate PTEN/AKT signaling, thus suppressing PC tumorigenesis.

## Conclusions

In summary, this is the first report that FLVCR1-AS1 is inhibited in PC and that low FLVCR1-AS1 expression is associated with a grave prognosis. Moreover, FLVCR1-AS1 was shown to act as a tumor suppressor gene in PC by inhibiting growth and metastasis both *in vitro* and *in vivo*. Additionally, FLVCR1-AS1 is a direct transcriptional target of KLF10 and could, in turn, increase KLF10 expression by relieving the inhibitory effects of miR-513c-5p or miR-514b-5p on KLF10 expression by acting as a ceRNA to sequester these miRNAs. Thus, our results suggest a model mechanism in which the FLVCR1-AS1/KLF10 positive feedback loop suppresses PC progression (Fig. 7F). Our findings provide novel insights into the multiplicity of lncRNA-miRNA-mRNA interactions and offer promising strategies for novel targeted PC therapies.

## Supplementary Information

**Additional file 1: Supplementary Figure S1.** Prognostic analysis of miR-513c-5p and miR-514b-5p using survival data from TCGA.

**Additional file 2: Supplementary Figure S2.** Potential target mRNAs of miR-513c-5p and miR-514b-5p were predicted using RAID, miRDB, miRTarBase and TargetScan.

**Additional file 3: Supplementary Figure S3.** The tumor suppression effects of FLVCR1-AS1 depend on KLF10. (A) KLF10 expression levels in 77 PC tissues compared to matched normal tissues. (B) Prognostic analysis of KLF10 using survival data of 51 patients from our center. (C) IHC staining scores of KLF10 expression in 25 paired PC samples. Representative images of different KLF10 expression levels are shown in the left panel, original magnification: ×40. (D) KLF10 expression was elevated in PC tissues with relatively high FLVCR1-AS1 expression levels, original magnification: ×40. (E) Expression of cell cycle-related proteins and metastasis-related proteins with indicated treatment were evaluated by western blotting. (F-G) CCK-8 and EdU assays suggested that FLVCR1-AS1 overexpression inhibits PANC-1 cell proliferation. Knockdown of KLF10 promotes PANC-1 cell proliferation. Co-transfection with si-KLF10 and pcDNA-FLVCR1-AS1 eliminated the decrease in proliferation rates. (H-I) Wound healing and transwell assays showed that FLVCR1-AS1 overexpression decreases PANC-1 cell migration. KLF10 silencing promotes PANC-1 cell migration. Co-transfection with si-KLF10 and pcDNA-FLVCR1-AS1 abolished the decrease in migration abilities. Scale bar = 50 μm *P < 0.05; **P < 0.01; ***P < 0.001; ns, no significance. All experiments were repeated three times.

**Additional file 4: Table S1. **miRNA mimics sequences used in this study.

**Additional file 5: Table S2. **siRNA sequences used in this study.

**Additional file 6: Table S3.** Primer sequences used in this study.

**Additional file 7: Table S4. **Antibodies used for western blotting.

## Data Availability

The datasets supporting the conclusions of this article are included within the article and its additional files.

## Abbreviations

PC: Pancreatic cancer
lncRNA: Long non-coding RNA
ceRNA: Competitive endogenous RNA
qRT-PCR: Quantitative reverse transcription polymerase chain reaction
KLF10: Kruppel-like factor 10
HPNE: Human normal pancreatic ductal epithelial cells
NC: Negative control
RIP: RNA immunoprecipitation
FISH: Fluorescent in situ hybridization
3’UTR: 3′-untranslated regions
CCK-8: Cell count kit-8
EdU: 5-ethynyl-20-deoxyuridine
AGO2: Argonaute-2
IHC: Immunohistochemistry
HE: Hematoxylin–eosin
ChIP: Chromatin immunoprecipitation
SD: Standard deviation
