# Supplementary figures and images for: Positive feedback between lncRNA FLVCR1-AS1 and KLF10 may inhibit pancreatic cancer progression via the PTEN/AKT pathway
Source: J Exp Clin Cancer Res. 2021 Oct 11;40:316. doi: 10.1186/s13046-021-02097-0 (PMC8507233; doi:10.1186/s13046-021-02097-0)

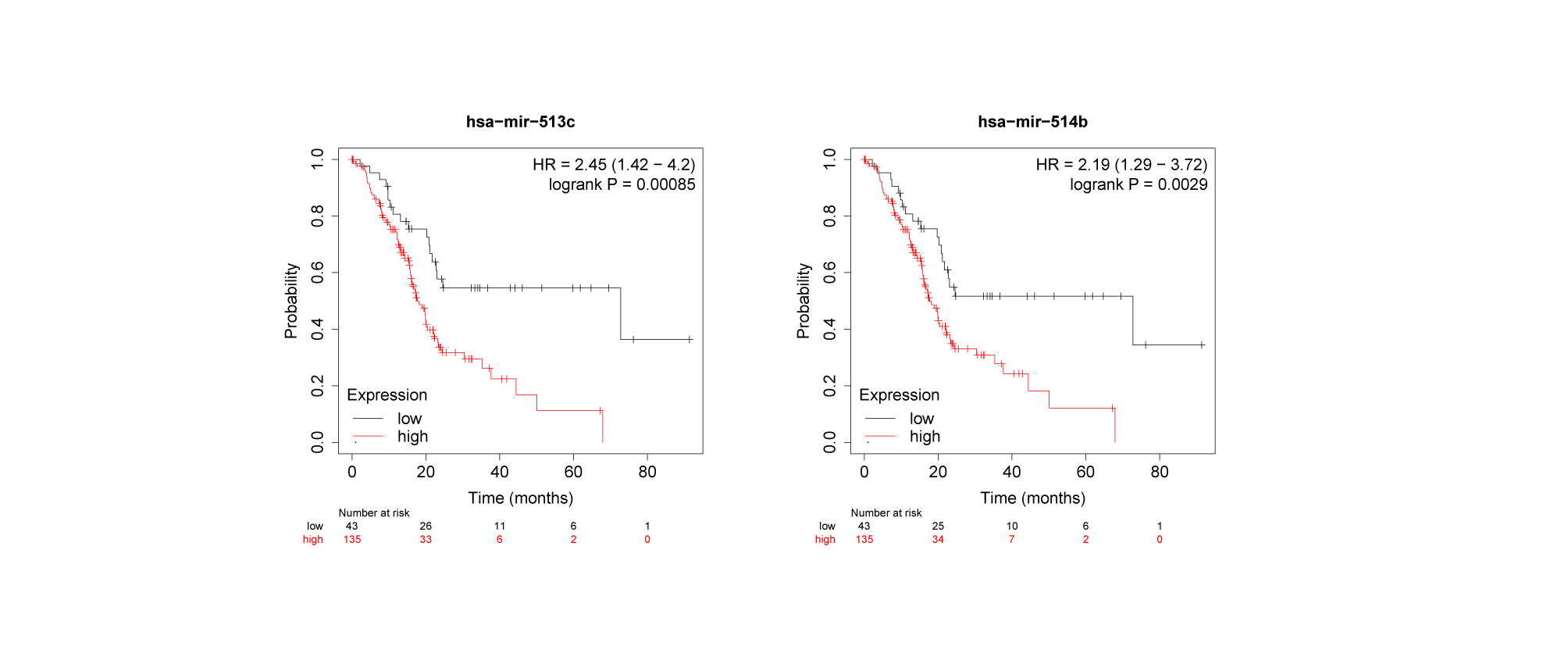

Supplement: Supplementary file 1 — Additional file 1: Supplementary Figure S1. Prognostic analysis of miR-513c-5p and miR-514b-5p using survival data from TCGA. [file 13046_2021_2097_MOESM1_ESM.tif]

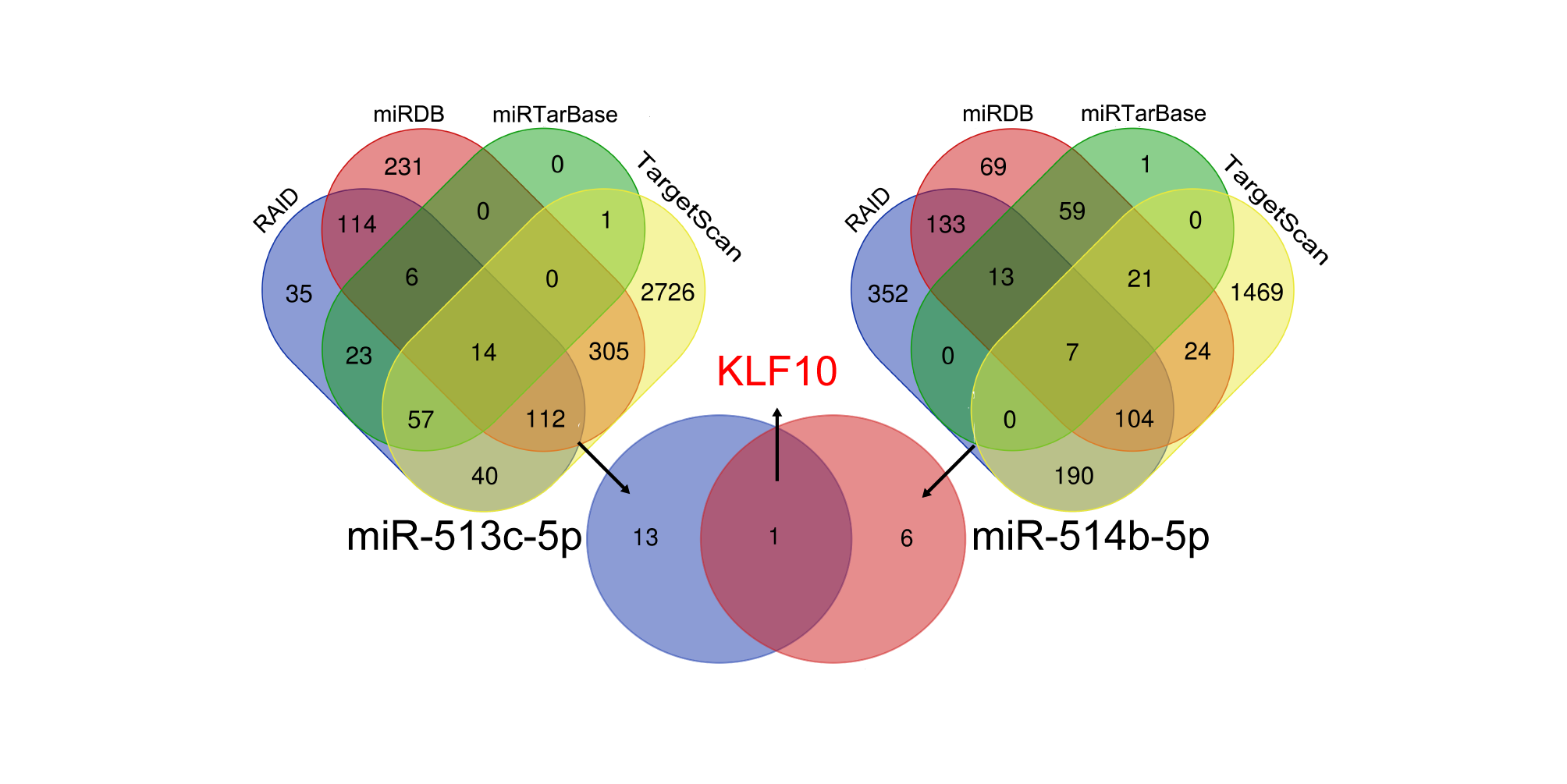

Supplement: Supplementary file 2 — Additional file 2: Supplementary Figure S2. Potential target mRNAs of miR-513c-5p and miR-514b-5p were predicted using RAID, miRDB, miRTarBase and TargetScan. [file 13046_2021_2097_MOESM2_ESM.tif]

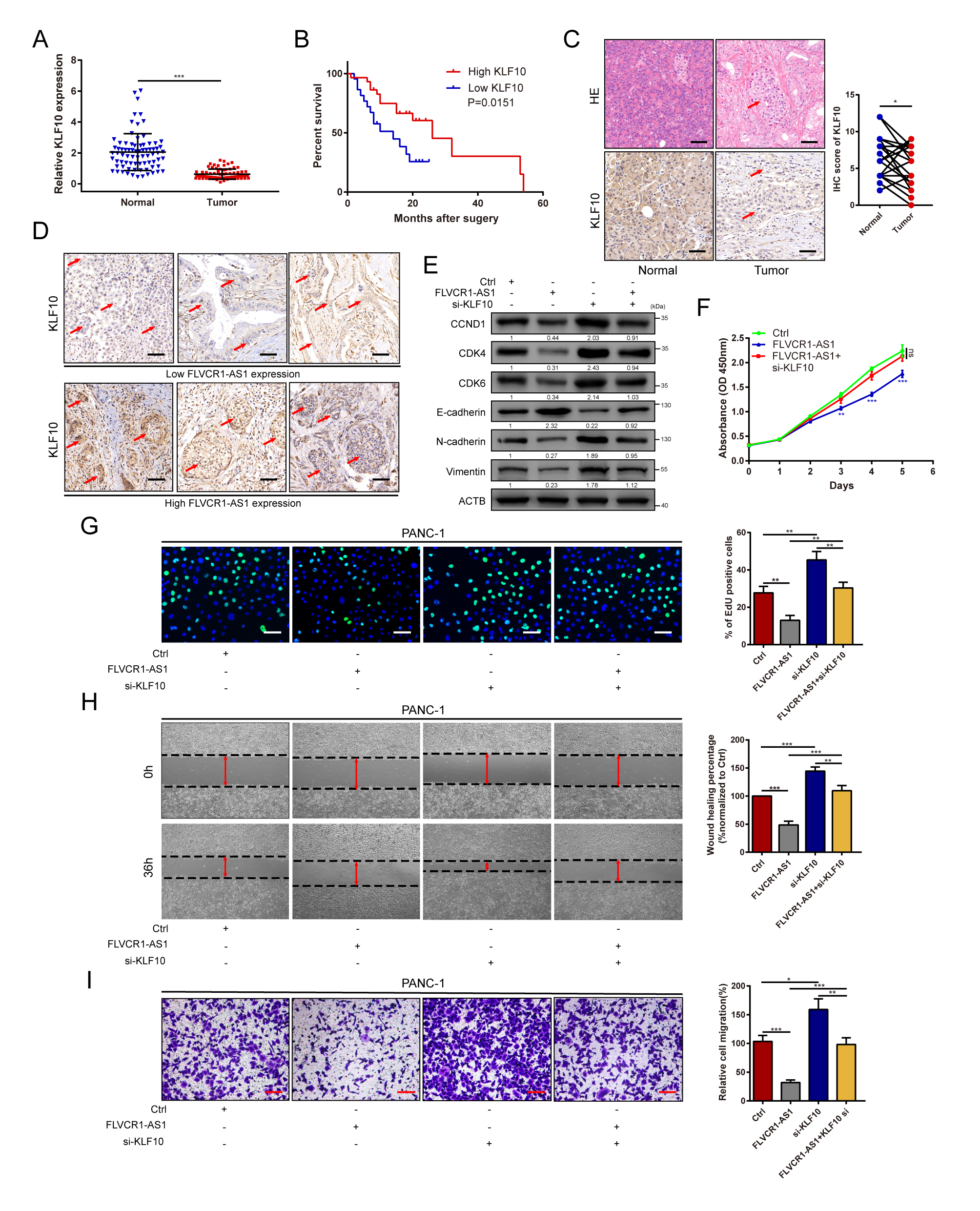

Supplement: Supplementary file 3 — Additional file 3: Supplementary Figure S3. The tumor suppression effects of FLVCR1-AS1 depend on KLF10. (A) KLF10 expression levels in 77 PC tissues compared to matched normal tissues. (B) Prognostic analysis of KLF10 using survival data of 51 patients from our center. (C) IHC staining scores of KLF10 expression in 25 paired PC samples. Representative images of different KLF10 expression levels are shown in the left panel, original magnification: ×40. (D) KLF10 expression was elevated in PC tissues with relatively high FLVCR1-AS1 expression levels, original magnification: ×40. (E) Expression of cell cycle-related proteins and metastasis-related proteins with indicated treatment were evaluated by western blotting. (F-G) CCK-8 and EdU assays suggested that FLVCR1-AS1 overexpression inhibits PANC-1 cell proliferation. Knockdown of KLF10 promotes PANC-1 cell proliferation. Co-transfection with si-KLF10 and pcDNA-FLVCR1-AS1 eliminated the decrease in proliferation rates. (H-I) Wound healing and transwell assays showed that FLVCR1-AS1 overexpression decreases PANC-1 cell migration. KLF10 silencing promotes PANC-1 cell migration. Co-transfection with si-KLF10 and pcDNA-FLVCR1-AS1 abolished the decrease in migration abilities. Scale bar = 50 μm *P < 0.05; **P < 0.01; ***P < 0.001; ns, no significance. All experiments were repeated three times. [file 13046_2021_2097_MOESM3_ESM.tif]
