# Supplementary material for: Positive feedback between lncRNA FLVCR1-AS1 and KLF10 may inhibit pancreatic cancer progression via the PTEN/AKT pathway
Source: J Exp Clin Cancer Res. 2021 Oct 11;40:316. doi: 10.1186/s13046-021-02097-0 (PMC8507233; doi:10.1186/s13046-021-02097-0)
Supplement: Supplementary file 4 — Additional file 4: Table S1. miRNA mimics sequences used in this study. [file 13046_2021_2097_MOESM4_ESM.docx]

**Table S1** miRNA mimics sequences used in this study

| miR-513c-5p sense | 5′-UUCUCAAGGAGGUGUCGUUUAU-3′ |
| --- | --- |
| miR-513c-5p anti-sense | 5′-AUAAACGACACCUCCUUGAGAA-3′ |
| miR-514b-5p sense | 5′- UUCUCAAGAGGGAGGCAAUCAU-3′ |
| miR-514b-5p anti-sense | 5′-AUGAUUGCCUCCCUCUUGAGAA-3′ |
| miR-NC sense | 5′-UUCUCCGAACGUGUCACGUTT-3′ |
| miR-NC anti-sense | 5′-ACGUGACACGUUCGGAGAATT-3′ |
