# Supplementary material for: Positive feedback between lncRNA FLVCR1-AS1 and KLF10 may inhibit pancreatic cancer progression via the PTEN/AKT pathway
Source: J Exp Clin Cancer Res. 2021 Oct 11;40:316. doi: 10.1186/s13046-021-02097-0 (PMC8507233; doi:10.1186/s13046-021-02097-0)
Supplement: Supplementary file 5 — Additional file 5: Table S2. siRNA sequences used in this study. [file 13046_2021_2097_MOESM5_ESM.docx]

**Table S2** siRNA sequences used in this study

| si-KLF10 sense | 5′-GAACCCUCUCAAGUGUCAAAUTT-3′ |
| --- | --- |
| si-KLF10 anti-sense | 5′- AUUUGACACUUGAGAGGGUUCTT -3′ |
| siRNA-NC sense | 5′-UUCUCCGAACGUGUCACGUTT-3′ |
| siRNA-NC anti-sense | 5′-ACGUGACACGUUCGGAGAATT-3′ |
