# Supplementary material for: Positive feedback between lncRNA FLVCR1-AS1 and KLF10 may inhibit pancreatic cancer progression via the PTEN/AKT pathway
Source: J Exp Clin Cancer Res. 2021 Oct 11;40:316. doi: 10.1186/s13046-021-02097-0 (PMC8507233; doi:10.1186/s13046-021-02097-0)
Supplement: Supplementary file 6 — Additional file 6: Table S3. Primer sequences used in this study. [file 13046_2021_2097_MOESM6_ESM.docx]

**Table S3** Primer sequences used in this study

| Primers used for qPCR: | |
| --- | --- |
| FLVCR1-AS1 forward | GAACGAGAGAGCCACCGAAACAG |
| FLVCR1-AS1 reverse | CACCGACAAGGACAGCGATGC |
| ACTB forward | CATGTACGTTGCTATCCAGGC |
| ACTB reverse | CTCCTTAATGTCACGCACGAT |
| miR-513c-5p forward | CGCGTTCTCAAGGAGGTGTC |
| miR-513c-5p reverse | AGTGCAGGGTCCGAGGTATT |
| miR-514b-5p forward | GCGTTCTCAAGAGGGAGGC |
| miR-514b-5p reverse | AGTGCAGGGTCCGAGGTATT |
| U6 forward | CTCGCTTCGGCAGCACA |
| U6 reverse | AACGCTTCACGAATTTGCGT |
| KLF10 forward | CTTCCGGGAACACCTGATTTT |
| KLF10 reverse | GCAATGTGAGGTTTGGCAGTATC |
| Primers used for ChIP |  |
| FLVCR1-AS1 promoter1 forward | TGCAGCTCCACGCTCTCCTTCC |
| FLVCR1-AS1 promoter1 reverse | GGGACCAGACAAGGGGTGACTG |
| FLVCR1-AS1 promoter2 forward | CAGGCTGGTCTCAGACTCCTG |
| FLVCR1-AS1 promoter2 reverse | GAGAGGTCATTGTGAACAGCTCC |
