# Supplementary material for: Positive feedback between lncRNA FLVCR1-AS1 and KLF10 may inhibit pancreatic cancer progression via the PTEN/AKT pathway
Source: J Exp Clin Cancer Res. 2021 Oct 11;40:316. doi: 10.1186/s13046-021-02097-0 (PMC8507233; doi:10.1186/s13046-021-02097-0)
Supplement: Supplementary file 7 — Additional file 7: Table S4. Antibodies used for western blotting. [file 13046_2021_2097_MOESM7_ESM.docx]

| **Table S4** Antibodies used for western blotting | | | | |
| --- | --- | --- | --- | --- |
| **Antibody** | **Company** | **Cat. No.** | **Species** | **Dilution** |
| KLF10 | Proteintech | 11881-1-AP | Rabbit | 1:1000 |
| CCND1 | ABclonal | A0310 | Rabbit | 1:1000 |
| CDK4 | ABclonal | A11136 | Rabbit | 1:1000 |
| CDK6 | ABclonal | A0106 | Rabbit | 1:1000 |
| ACTB | Cell signaling | 4970 | Rabbit | 1:1000 |
| GAPDH | Proteintech | 60004-1-Ig | Mouse | 1:1000 |
| E-cadherin | Cell signaling | 3195 | Rabbit | 1:1000 |
| N-cadherin | Cell signaling | 13116 | Rabbit | 1:2000 |
| vimentin | Cell signaling | 5741 | Rabbit | 1:1000 |
| PTEN | ABclonal | A19104 | Rabbit | 1:1000 |
| p-AKT | Cell signaling | 13038 | Rabbit | 1:1000 |
| PI3K | Proteintech | 20662-1-AP | Rabbit | 1:1000 |
